# Single-cell RNA sequencing in cancer research: discovering novel biomarkers and therapeutic targets for immune checkpoint blockade

**DOI:** 10.1186/s12935-023-03158-4

**Published:** 2023-12-08

**Authors:** Boyu Sun, Ziyu Xun, Nan Zhang, Kai Liu, Xiangqi Chen, Haitao Zhao

**Affiliations:** Department of Liver Surgery, State Key Laboratory of Complex Severe and Rare Diseases, Peking Union Medical College Hospital, Chinese Academy of Medical Sciences and Peking Union Medical College, No.1, Shuaifuyuan, Dongcheng District, Beijing, 100730 China

**Keywords:** Immune checkpoint blockade, Single-cell RNA sequencing, Tumor microenvironment, Biomarker, Therapeutic target

## Abstract

Immune checkpoint blockade (ICB) has become a promising strategy in treating advanced cancers, providing significant survival benefits for patients with various cancer types. However, among the vast population of cancer patients, only a small fraction are able to respond to and derive benefits from ICB therapy. Numerous factors contribute to the diminished efficacy of ICB, with the complex tumor microenvironment (TME) playing an important role. Therefore, comprehensively understanding the intricate composition of the TME is critical for elucidating the mechanisms that underlie distinct responses to ICB in patients. Single-cell RNA sequencing (scRNA-seq) is a novel technique that reveals gene expression profiles of individual cells, facilitating the investigation of TME heterogeneity at a high resolution and the identification of key cell subsets participating in the response to ICB. This review emphasizes the importance of scRNA-seq in studying ICB and summarizes recent findings in the discovery of biomarkers that predict ICB response and novel potential therapeutic targets for immunotherapy. These findings suggest future directions for the clinical implementation of cancer immunotherapy, facilitating further advancements in precision medicine.

## Background

The immune system monitors the human body and eradicates abnormal cells. This intricate surveillance system operates through a complex network of immune cells and signaling molecules to identify and eliminate foreign invaders. Immune checkpoints, located on the surface of immune cells, play an important role in preventing their overactivation and maintaining immune homeostasis. Despite the defense mechanisms, cancer cells can evade immune surveillance by multiple mechanisms, such as binding to the immune checkpoints, thereby suppressing the anti-tumor response.

Treatment selection for cancer patients depends on the clinical stage, usually assessed using the TNM classification system. For patients with unresectable tumors or those in advanced stages, systemic treatment is necessary to control the progression of the disease. Immunotherapy, emerging after chemotherapy and targeted therapy, represents a promising avenue for improving patient survival. Compared with other treatments, immunotherapy shows the advantages of long-lasting anti-tumor effects and reduced side effects, making it a significant modality for treating cancers [1, 2]. Immunotherapy activates the immune system to kill cancer cells. Immune checkpoint inhibitors (ICIs), which inhibit programmed cell death 1 (PD-1) or programmed cell death 1 ligand 1 (PD-L1) and cytotoxic T lymphocyte-associated protein 4 (CTLA-4), have shown success in treating many cancers. Nonetheless, most patients receiving ICIs do not experience survival benefits. While biomarkers such as PD-L1 expression [3], mismatch repair deficiency (dMMR) or microsatellite instability-high (MSI-H) [4], and tumor mutational burden (TMB) [5] are routinely utilized to identify people who may benefit from immune checkpoint blockade (ICB), these methods are not always accurate in predicting response to ICB in clinical practice. Therefore, it is essential to identify novel biomarkers that better predict response to ICB.

Current immunotherapy strategies mainly focus on activating CD8^+^ T cells, the major cytotoxic immune cells that kill cancer cells via the Fas-FasL pathway and the perforin/granzyme mechanism [6]. Nonetheless, the tumor microenvironment (TME) is composed of heterogeneous cells, extracellular matrix (ECM) components, blood vessels, and biomolecules [7], and the complex network formed by direct and indirect interactions between these heterogeneous cells can contribute to an immunosuppressive pattern in the TME and result in ICB failure. Therefore, it is also essential to search for new therapeutic targets in the TME that can synergize with current ICIs to suppress tumors.

**Fig. 1:**
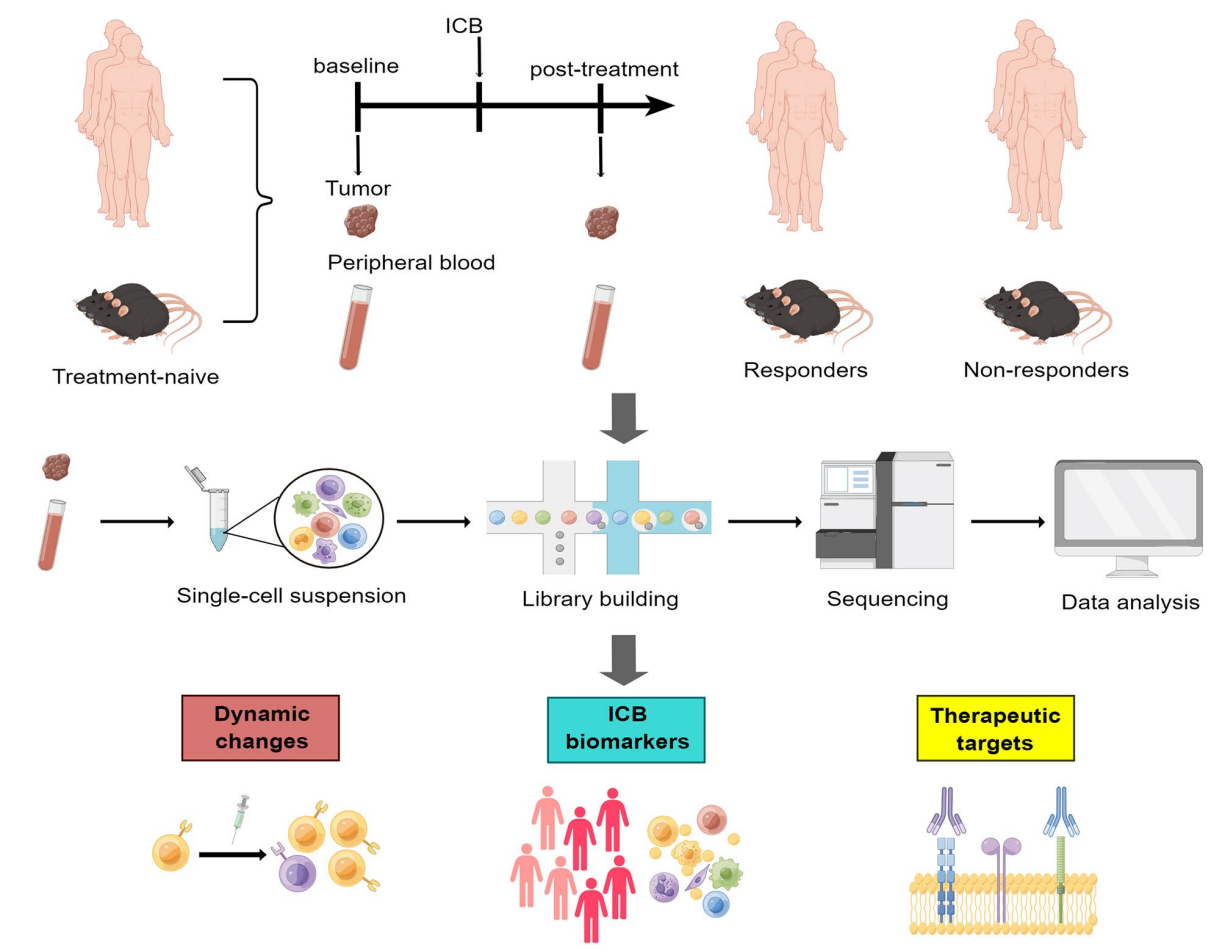
The schematic diagram of the application of scRNA-seq in ICB. Tumor or peripheral blood mononuclear cells are obtained from animal models or human patients and are first dissociated into single-cell suspensions. The cells are then lysed for reverse transcription and amplification to construct the cDNA library. The resulting scRNA-seq data can be applied to study the dynamic changes in the TME or peripheral blood during ICB therapy, discover novel biomarkers predictive of ICB response, and search for potential therapeutic targets. scRNA-seq, single-cell RNA sequencing; ICB, immune checkpoint blockade. Created with Figdraw (www.figdraw.com)

Single-cell technologies have the capacity to unravel the phenotypes of individual cells within a tissue, revolutionizing our understanding of cancer biology. Single-cell RNA sequencing (scRNA-seq), involving the main steps of single-cell isolation, cell lysis for mRNA capture, reverse transcription, cDNA amplification, and sequencing library construction, enables the researchers to decipher the cell heterogeneity. Unlike bulk RNA sequencing (RNA-seq), which quantifies the average expression of genes in a variety of cells, scRNA-seq allows for the detection of transcriptomic heterogeneity by measuring RNA expression levels of individual cells [8]. This technology can detect heterogeneity in various samples, including cancer and peritumoral tissues, as well as peripheral blood, enabling the discovery of new cell subsets with unique gene expression profiles and the studies of intercellular crosstalk, cellular differentiation trajectories, and gene regulation networks within the TME [9–11]. Therefore, scRNA-seq is a potent tool for unraveling why certain patients respond to ICB while others do not. By revealing differences in cellular components between responders and non-responders, and by discovering pivotal cell subsets that contribute to the response to ICB, scRNA-seq can identify novel biomarkers that have the potential to predict ICB responsiveness and new molecules that can be therapeutically targeted. In this review, we will first introduce the present status of ICB therapy and briefly overview the immunosuppressive cellular components in the TME. We will then review recent scRNA-seq studies that reveal the dynamic changes in cellular components during ICB treatment, discover novel biomarkers, and identify new therapeutic targets (Fig. 1).

## Immune checkpoint blockade and predictive biomarkers in cancer treatment

In current clinical practice, cancer staging is essential for disease assessment and treatment selection. The TNM system is the most widely used classification method which evaluates the primary tumor (T), regional lymph nodes (N), and distant metastasis (M) [12]. In addition, many cancers also have their unique staging systems, such as the Barcelona Clinic Liver Cancer (BCLC) staging system in hepatocellular carcinoma (HCC) [13]. Regrettably, many patients are diagnosed at advanced stages and fail to undergo surgery, which is the only radical treatment method. Consequently, other therapeutic approaches, including radiotherapy, chemotherapy, targeted therapy, and immunotherapy, have become necessary for the management of advanced-stage tumors. Among these methods, radiotherapy is a local treatment modality that employs high-energy radiation to eradicate cancer cells within localized tumors. It has limited impacts on distant lesions, and it can cause damage to healthy tissues, leading to toxicity [14]. Chemotherapy is a first-line treatment for many cancers, functioning by inhibiting the proliferation of cancer cells. However, it cannot effectively differentiate between cancer cells and normal cells and can cause various side effects [15]. Targeted therapy precisely targets specific proteins or signal transduction pathways in cancer cells, taking effect quickly with low toxicity. However, it is effective mainly for patients with specific genetic mutations [16].

Immunotherapy, mainly including immune checkpoint inhibitors, cancer vaccines, and CAR-T cell therapy, is the latest emerging therapeutic approach that has demonstrated promising clinical efficacy [17]. Unlike other treatment modalities, immunotherapy activates the patient’s immune system instead of directly targeting cancer cells, thereby leading to long-lasting clinical benefits.

### Currently used immune checkpoints and their inhibitors

In the cancer immune cycle, antigen-presenting cells (APCs) capture tumor antigens and then present them to naïve T cells when migrating to the lymph nodes. Then the T cells are primed and activated, obtaining the ability to eliminate cancer cells [18]. However, during this cycle, immune checkpoints function as a brake to suppress T cell activation, thereby enabling the immune escape of cancer cells. The immunoinhibitory receptors PD-1 and CTLA-4 are the most-studied immune checkpoints regulating the activation of T cells. Targeting these receptors with antibodies can activate effector T cells in suppressed states and enhance the immune response, which has yielded encouraging outcomes in cancer treatment.

#### CTLA-4 and PD-1/PD-L1

CTLA-4 is present on the surface of T cells and binds B7-1 (CD80) and B7-2 (CD86), competing with the costimulatory receptor CD28. CTLA-4 inhibition was shown to induce potent immune responses and tumor-killing activity of T cells in vivo [19]. Ipilimumab, the first immune checkpoint inhibitor targeting CTLA-4, was approved by the US Food and Drug Administration (FDA) in 2011 after showing improved survival benefits in patients with metastatic melanoma [20]. PD-1 is another immune checkpoint that binds to the ligands PD-L1 and PD-L2, inducing the suppression of T cell receptor (TCR)-mediated proliferation and the CD28-mediated costimulation pathway [21, 22]. Inhibiting the interaction between PD-1 and PD-L1 can enhance the anti-tumor responses of T cells. The first anti-PD-1 antibody, nivolumab, and the first anti-PD-L1 antibody, atezolizumab, demonstrated their anti-tumor efficacy in clinical trials and were approved by the FDA in 2014 and 2016, respectively [23, 24].

Currently, the FDA has approved several anti-PD-1/PD-L1 and anti-CTLA-4 drugs for the treatment of various cancers. These ICIs have shown anti-tumor efficacy in treating cancers, including HCC, melanoma, renal cell carcinoma (RCC), triple-negative breast cancer (TNBC), non-small cell lung carcinoma (NSCLC), head and neck squamous cell carcinomas (HNSCC), MSI-H/dMMR colorectal cancer (CRC), urothelial cancer, and Merkel cell carcinoma (MCC), as well as nonsolid tumors, such as diffuse large B-cell lymphoma and classic Hodgkin lymphoma [25]. However, the clinical efficacy varies greatly among patients receiving ICIs, and only a fraction of patients receiving ICB achieve objective responses, with a response rate of only 20-30% on average [26]. Some patients may respond well to ICB and even achieve a complete response, while more patients fail to benefit from treatment or only show a temporary response. In addition, the response rate to ICB varies across different cancer types, with an objective response rate of 87.1% in patients with classical Hodgkin lymphoma [27] and no more than 30% in patients with other cancers, such as melanoma, RCC, NSCLC, and HCC [28, 29].

#### Other immune checkpoints

In addition to the canonical immune checkpoints PD-1 and CTLA-4, other immune checkpoint molecules have been discovered, such as lymphocyte-activation gene 3 (LAG-3), T-cell immunoglobulin and mucin-domain 3 (TIM-3), and T cell immunoreceptor with Ig and ITIM domains (TIGIT). LAG-3 is primarily expressed on activated T cells and CD4^+^ regulatory T cells (Tregs) and can bind to stable peptide-MHC class II complexes, downregulating T-cell activation [30]. LAG-3 can inhibit the immune response synergistically with PD-1, acting as a nonredundant immune checkpoint molecule [31]. TIM-3 is expressed on activated CD8^+^ T cells, CD4^+^ T helper 1 (Th1) cells, Tregs, monocytes, and natural killer (NK) cells [32]. By binding to ligands such as galectin-9, TIM-3 triggers apoptosis of CD8^+^ T cells, negatively regulating the anti-tumor activity [33]. And a negative correlation has been found between TIM-3 expression on PD-1^+^ CD8^+^ T cells and clinical outcomes in cancer patients [34]. TIGIT is another immune checkpoint protein showing expression on activated T cells, Tregs, and NK cells [35]. It competes with the costimulatory receptor CD226 for the binding of CD155, which results in T and NK cell inhibition, similar to the CD28/CTLA-4 axis [35, 36]. Additionally, TIGIT can bind to CD226 directly and prevent CD155/CD226 signaling by disrupting CD226 homodimerization [35, 36]. Numerous novel drugs targeting these immune checkpoints are currently undergoing clinical trials, either alone or combined with PD-1/PD-L1 inhibitors. Some trials have reported promising results, suggesting that these drugs might enhance existing immunotherapy.

### Biomarkers used in current clinical practice for ICB

The application of ICIs in cancer treatment has shown substantial success. However, there is significant variability in outcomes among patients treated with ICB, and the overall response rate remains relatively low [26]. To prevent non-responsive patients from undergoing ineffective treatments and incurring high costs, it is imperative to find reliable biomarkers to identify patients who may benefit from ICB. Three biomarkers are often used to identify potential ICB responders in current clinical practice, including PD-L1, TMB, and MSI-H/dMMR, and they have been found to be associated with the efficacy of ICB in clinical practice [3–5]. However, these biomarkers are not perfect, as they face inevitable challenges in practical applications. Their predictive abilities vary across different cancer types, and standardized thresholds to discriminate patients are lacking [37]. Furthermore, these biomarkers cannot reflect the heterogeneity of the patient’s TME, which directly influences the anti-tumor response. Arbitrarily relying on these biomarkers for discrimination may deprive certain patients of the opportunity to benefit from ICB.

#### PD-L1

PD-L1 is often expressed on the surface of cancer cells or APCs under conditions of T cell infiltration and interferon-γ production, which indicates the preexistence of T cell response and predicts the response when receiving ICIs [38]. PD-L1 is the first FDA-approved biomarker for ICB, and its expression in tumor tissues is primarily detected by immunohistochemistry in clinical practice. A high tumor PD-L1 expression level predicts enhanced ICB efficacy [3], and higher expression of PD-L1 is associated with a more substantial improvement [39]. However, some patients with low PD-L1 expression can also achieve survival benefits from ICB [40, 41]. Conversely, some patients with high PD-L1 expression may not respond to ICB, as the PD-1/PD-L1 interaction is more correlated with ICB response compared with PD-L1 expression levels [42]. PD-L1 expression exhibits temporospatial dynamics as it is heterogeneous across different tissue sample sites and at different time points, which may also compromise its predictive accuracy [43, 44]. Moreover, researchers have discovered that specific somatic molecular alterations were also correlated with ICB outcome, which could also differentially impact the predictive power of PD-L1 [43]. In addition, the predictive ability of PD-L1 is diminished when ICB is combined with other therapies such as chemotherapy [45]. These limitations indicate that PD-L1 expression is an imperfect biomarker, emphasizing the urgent need for other biomarkers.

#### TMB

TMB represents the number of non-synonymous somatic mutations per million bases of a tested genomic sequence, which is often determined by whole-exome sequencing or targeted panel sequencing [46]. A higher TMB may lead to a greater mutational load, increase the generation of immunogenic neoantigens, and activate more CD8^+^ T cells, thus enhancing the response to ICB. The KEYNOTE-158 study showed significantly enhanced ICB outcomes in TMB-high patients compared with TMB-low patients with advanced solid tumors [47]. Patients with high TMB tend to benefit from ICB irrespective of PD-L1 expression, indicating TMB as a nonredundant biomarker [48]. However, a higher TMB does not necessarily mean a better response to ICB. Only a small fraction of somatic mutations can be transcribed and translated into neoantigens that are presented by major histocompatibility complex (MHC) molecules on the cell surface. And even fewer neoantigens can be recognized by the immune system, thereby leading to limited recruitment of CD8^+^ T cells [49]. A pan-cancer study [50] found that high TMB did not accurately predict ICB response in certain cancers that showed no correlation between the levels of CD8^+^ T cells and neoantigen loads, and some TMB-high patients even exhibited worse ICB outcomes in these cancer types. In addition, the diversity of human leukocyte antigen class I (HLA-I) genotypes is also associated with ICB response, which influences the presentation of neoantigens to CD8^+^ T cells [51]. Therefore, a high TMB does not ensure the production of adequate immunogenic neoantigens recognized by CD8^+^ T cells and may not always predict the response to ICB.

#### MSI-H/dMMR

Loss-of-function mutations or transcriptional silencing of mismatch repair genes like *MLH1*, *MSH2*, *MSH6*, and *PMS2* can alter the size of DNA microsatellite regions and is defined as microsatellite instability (MSI) [52]. MSI-H is a specific type of TMB-high, as most MSI-H tumors show high TMB while only a small fraction of TMB-high tumors are MSI-H [53]. MSI-H or dMMR can increase the TMB and generate immunogenic neoantigens, which can serve as a useful biomarker predicting a favorable ICB response [4]. However, the frequency of MSI varies significantly across different types of cancers. MSI-H/dMMR is more common in endometrial cancer, gastric cancer, and colorectal cancer, while it is rare in other cancers, such as lung adenocarcinoma, limiting its utility as a pan-cancer biomarker [54, 55].

#### Other biomarkers

While the biomarkers discussed above have varying degrees of limitations, it is imperative to unravel the underlying mechanisms and develop robust predictive models to better stratify patients. Recent studies have identified other factors that may predict the response to ICB, including specific infiltrating immune cells in the TME [56], specific gene mutations such as *BRCA2* [57], somatic copy number alterations [58], circulating tumor DNA (ctDNA) [59], and the gut microbiome [60]. Owing to the heterogeneity of the TME, the predictive accuracy of biomarkers may be impacted by specific cell subclusters. Recent advances in single-cell technologies such as scRNA-seq have provided a detailed investigation of cellular components in the TME and peripheral blood to discover critical cell subsets that correlate with the response to ICB.

## Cellular components of the tumor microenvironment

**Fig. 2:**
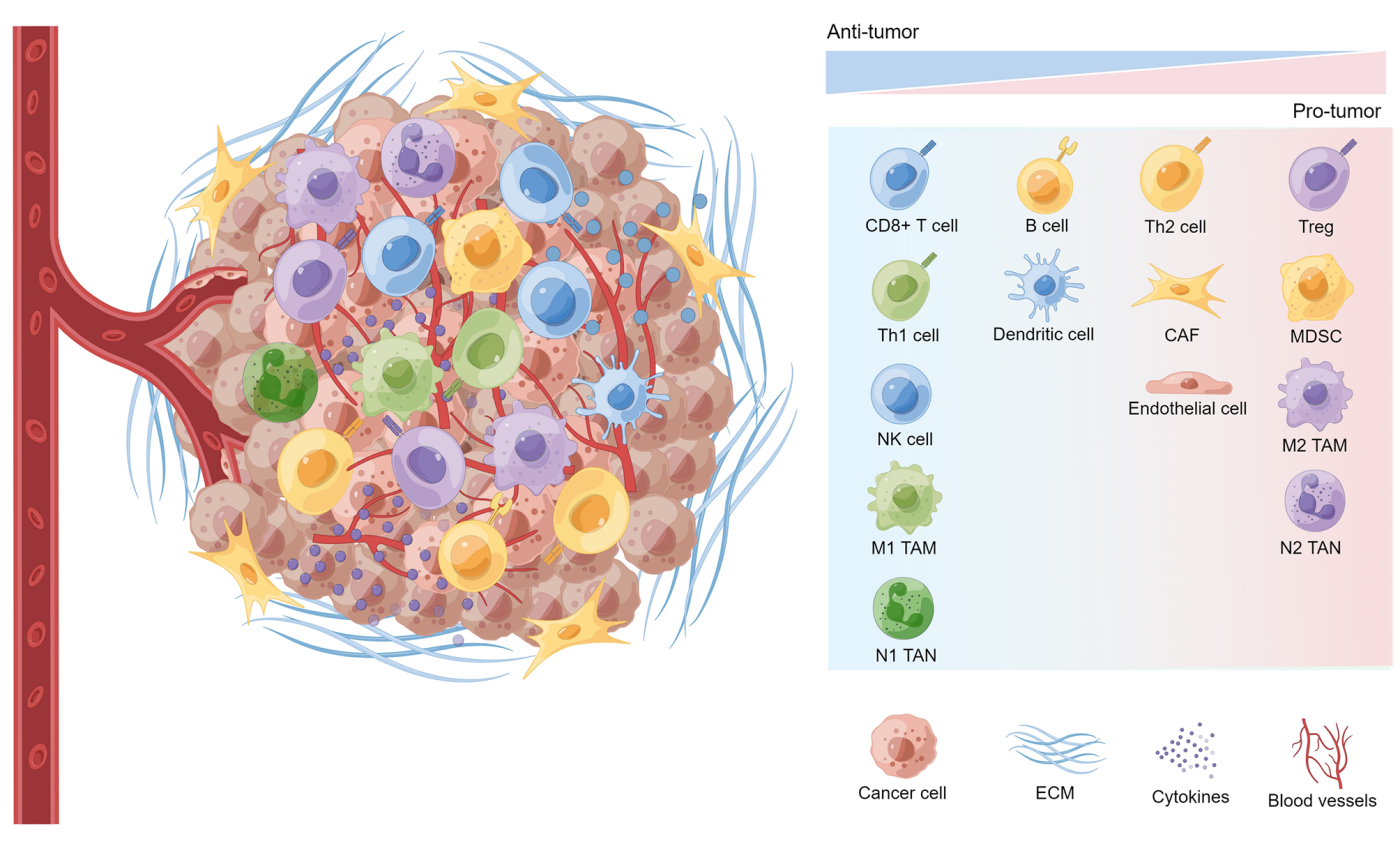
The schematic presentation of the TME composition. The tumor microenvironment (TME) contains both cellular and non-cellular components. Multiple cell types are present in the TME, including cancer cells and stromal cells. The heterogeneous cells contain both immunostimulatory and immunosuppressive subsets that exert anti-tumor and pro-tumor effects, respectively. And the preponderance of the pro-tumor cell subsets contributes to the immunosuppressive TME, leading to cancer progression and the failure of ICB. The application of single-cell RNA sequencing (scRNA-seq) provides insights into TME heterogeneity. Th1 cell, CD4^+^ T helper 1 cell; NK cell, natural killer cell; TAM, tumor-associated macrophage; TAN, tumor-associated neutrophil; Th2 cell, CD4^+^ T helper 2 cell; CAF, cancer-associated fibroblast; Treg, CD4^+^ regulatory T cell; MDSC, myeloid-derived suppressor cell; ECM, extracellular matrix. Created with Figdraw (www.figdraw.com)

Mobilizing immune cells to induce a durable anti-tumor immune response is the primary goal of ICB. The TME is a heterogeneous microenvironment including cancer cells and stromal cells that can either promote or suppress anti-tumor effects and influence cancer cell growth, metabolism, proliferation, and metastasis (Fig. 2). Innate immune cells, including dendritic cells (DCs), NK cells, macrophages, natural killer T (NKT) cells, and gamma delta T (γδ T) cells, participate in killing cancer cells in a nonspecific manner [61]. Anti-tumor adaptive immune cells include B cells and T cells, with the latter comprising CD8^+^ cytotoxic T cells and CD4^+^ helper T cells. Despite the existence of anti-tumor immune cells, the TME overall exhibits a pro-tumor pattern due to the presence of immunosuppressive cells, including Tregs, myeloid-derived suppressor cells (MDSCs), tumor-associated macrophages (TAMs), tumor-associated neutrophils (TANs), regulatory B cells (Bregs), and cancer-associated fibroblasts (CAFs) [62, 63]. These immunosuppressive components of the TME may impede the activation of anti-tumor immunity and contribute to ICB failure.

### CD8^+^ T cells

Within T cells, CD8^+^ T cells are the primary effector cells to kill cancer cells, and CD4^+^ T cells, including Tregs and helper T cells, modulate the function of CD8^+^ T cells. CD8^+^ T cells exert tumor-killing cytotoxicity primarily through two mechanisms: the Fas-FasL pathway and the perforin-granzyme pathway [6]. In the Fas-FasL pathway, CD8^+^ T cells can secrete vesicles containing the Fas ligand (FasL) that bind to the Fas molecule on cancer cells, activating intracellular caspase 8 and inducing cell apoptosis. In the perforin-granzyme pathway, perforin released by CD8^+^ T cells can open channels on the surface of cancer cells, allowing granzymes to enter and lead to apoptosis.

### Tregs

Tregs are a subset of immunosuppressive CD4^+^ T cells that express forkhead box protein P3 (FOXP3) and participate in maintaining self-tolerance [64]. Tregs are more abundant in tumor tissues than in peripheral blood and peritumoral tissues, suggesting their pro-tumor function [65]. FOXP3^+^ CD4^+^ T cells can be classified into effector Treg cells (eTregs), naïve Treg cells, and non-Treg cells based on specific markers. Of these subsets, eTregs (CD4^+^ CD25^high^ FOXP3^high^ CD45RA^−^) are the predominant subset of Tregs that can differentiate from naïve Treg cells upon TCR stimulation and exert potent immunosuppressive effects [66]. Tregs facilitate immunosuppression via multiple mechanisms, including consumption of interleukin-2 (IL-2), suppression of APC function, production of immunosuppressive molecules such as IL-10 and transforming growth factor-β (TGF-β), and destruction of effector cells [66]. 4-1BB is specifically expressed on activated intratumoral Tregs, enhancing their suppressive function [67].

### Macrophages

Macrophages exhibit different developmental trajectories depending on the context and are commonly classified into two classes: classically activated macrophages (M1) and alternatively activated macrophages (M2). M1 macrophages express CD80, CD86, and HLA-DR surface markers and have anti-tumor effects by producing molecules such as IL-12, reactive oxygen species (ROS), and nitric oxide synthase (iNOS). In contrast, M2 macrophages express CD163, CD206, and CD204 surface markers and exert pro-tumor effects by producing IL-10, vascular endothelial growth factor (VEGF), and matrix metalloprotein (MMP) to promote angiogenesis, matrix remodeling, and immunosuppression [68, 69]. The TAM population is heterogeneous and primarily exhibits an M2-like immunosuppressive pattern. Nonetheless, the binary M1/M2 division of macrophages is imprecise, as recent scRNA-seq studies have identified specific TAM clusters that express both M1 and M2 signatures [70, 71] and discovered TAM clusters that do not fall within the conventional M1/M2 classification [72], highlighting the complexity of TAMs and the need for further investigation. Additionally, scRNA-seq can identify novel TAM subsets with crucial effects. FOLR2^+^ macrophages, a subset of tissue-resident macrophages, can prime CD8^+^ T cells, leading to a better prognosis [73]. Conversely, TAMs enriched for the scavenger receptor MARCO in gliomas are associated with the mesenchymal signature and hypoxia, contributing to tumor progression [74]. TAMs highly expressing *SPP1* can drive malignancy and are correlated with unfavorable outcomes and metastasis in CRC [75].

### Neutrophils

Neutrophils present in the TME exhibit heterogeneity and can be classified into anti-tumor N1-TANs and pro-tumor N2-TANs, similar to TAMs [76]. N2-polarized TANs constitute the major part of TANs. Cytokines such as TGF-β can facilitate the transition of TANs from the N1 to the N2 phenotype, promoting tumor cell proliferation, matrix remodeling, and angiogenesis [76]. Recent studies using scRNA-seq have further investigated the heterogeneity within TAN subtypes. In pancreatic ductal adenocarcinoma (PDAC), a terminally differentiated TAN subcluster named TAN-1 highly expresses VEGFA and exhibits hyperactivated glycolytic activity, which is correlated with poor prognosis [77]. In liver tumors, scRNA-seq analysis identified three pro-tumor TAN subsets (CCL4^+^ TANs, IFIT1^+^ TANs, and SPP1^+^ TANs) and one anti-tumor subset (APOA2^+^ TANs) [78]. CCL4^+^ TANs secrete CCL4 protein and recruit macrophages via the CCL4-CCR5 axis, leading to immunosuppression in the TME. IFIT1^+^ TANs highly express *CD274* (encoding PD-L1) and directly suppress the function of CD8^+^ T cells.

### CAFs

CAFs are another important constituent of the TME and are particularly abundant in tumors enriched for tumor reactive stroma, such as cholangiocarcinoma, pancreatic cancer, and breast cancer [79]. CAFs originate primarily from tissue-resident fibroblasts, and they are also derived from fibrocytes, epithelial cells, mesenchymal stem cells, and endothelial cells. In some rare conditions, CAFs may also be derived from the transdifferentiation of adipocytes, pericytes, and smooth muscle cells [80]. CAFs participate in ECM remodeling by producing ECM components or matrix metalloproteinases (MMPs). They also engage in crosstalk with other cells and contribute to angiogenesis and cancer cell metastasis by secreting multiple cytokines, including VEGF, TGF-β, IL-6, C-X-C motif chemokine ligand 12 (CXCL12), and C-C motif chemokine ligand 2 (CCL2) [80, 81]. However, CAFs are a heterogeneous population including both pro- and anti-tumor subsets. Two important subsets of CAFs have been identified: inflammatory CAFs (iCAFs) and myofibroblastic CAFs (myCAFs), which are characterized by cytokine secretion and stromal remodeling, respectively [82]. Some scRNA-seq studies have confirmed the existence of these two CAF subsets and identified other novel CAF subclusters in various cancer types [83–86].

### Endothelial cells

Endothelial cells (ECs) of blood vessels in the tumor control the recruitment of immune cells by producing angiocrine factors [87]. ECs also control the intravasation and extravasation of cancer cells, resulting in tumor progression and metastasis [87]. One scRNA-seq study identified a tumor-specific PLVAP^+^ EC subset that could be activated by VEGF signaling and contribute to the immunosuppressive TME [88]. However, ECs can also exert anti-tumor effects, as a study found that MECA-79^+^ tumor-associated HEV endothelial cells could mediate lymphocyte extravasation during ICB and enhance anti-tumor immunity [89].

## Dynamic changes during ICB therapy revealed by scRNA-seq

Reprogramming of the TME is a key outcome of ICB therapy. To understand the mechanisms underlying ICB function, it is essential to capture the dynamic changes in cellular components of the TME or peripheral blood before and after treatment.

As CD8^+^ T cells are the predominant cells responsible for tumor eradication that experience a series of lineage transitions and transcriptional changes during the anti-tumor response, it is especially important to study the changes in CD8^+^ T cells. CD8^+^ T cells are heterogeneous and multiple states along the development trajectory have been defined by recent studies. Some scRNA-seq studies have identified a CD8^+^ T cell development trajectory starting with *LEF1*^+^ naïve cells, transitioning through *GZMK*^+^ transitory cells, and then bifurcating into two branches with two distinct final states: *CX3CR1*^+^ terminally differentiated effector memory or effector T cells (Temra) and *LAYN*^+^ exhausted T cells (Tex) [90–92]. Notably, exhaustion is a gradual process, and there are different subsets of exhausted CD8^+^ T cells. Two subsets, progenitor exhausted T cells (Tex^prog^) and terminally exhausted T cells (Tex^term^), have different expression profiles and transcriptional changes during ICB [93]. Transcription factor 1 (TCF1), encoded by *TCF7*, is a crucial transcription factor that maintains stem-like functions of CD8^+^ progenitor cells, and the absence of Tcf1 indicates progressive differentiation into exhausted CD8^+^ T cells [94]. TCF-1^+^ PD-1^+^ Tex^prog^ cells show stem-like signatures, have relatively lower levels of cytotoxicity, and can differentiate into Tex^term^ cells. In contrast, TCF-1^−^ PD-1^+^ Tex^term^ cells are more exhausted, express higher levels of immune checkpoints, and transiently exhibit robust tumor-killing activity by producing more cytotoxic cytokines before rapidly becoming dysfunctional [93, 95, 96]. Of the two Tex subsets, Tex^prog^ cells are ICB-responsive, and Tex^term^ cells are not [93, 97]. This is because Tex^prog^ cells can both self-renew and differentiate into Tex^term^ cells to temporarily fight against cancer cells, while Tex^term^ cells cannot proliferate. These findings reveal that CD8^+^ T cells contain multiple subsets along the activation and exhaustion trajectory. Understanding the dynamic subsets and states of CD8^+^ T cells is crucial for elucidating the mechanisms underlying studying immunotherapeutic strategies against cancer.

Use of scRNA-seq can capture the changes among different CD8^+^ T cell subsets during ICB. Integration of TCR sequencing (TCR-seq) with scRNA-seq provides insights into CD8^+^ T cell clonality, diversity, and dynamics in both human and animal models [98].

ScRNA-seq studies have found that CD8^+^ T cells transitioned from a naïve or stem state to an active or effector state during ICB. Zhou et al. [99] found four major CD8^+^ T cell subclusters in murine oral carcinoma, including Tcf7^+^ Pd1^−^ naïve/memory cells, Tcf7^+^ Pd1^+^ progenitor cells, Tcf7^−^ Pd1^+^ effector cells, and Mki67^+^ proliferative cells. They demonstrated the transition from naïve/memory and progenitor cells to effector cells and the acquisition of a more activated/exhausted state during ICB. Kurtulus et al. [100] studied PD-1^−^ CD8^+^ T cells from mice bearing colon carcinoma and observed a reduction in naïve-like cells along with the expansion of memory-precursor- and effector-like cells after anti-PD-1 and anti-TIM-3 blockade. Similarly, Khojandi et al. [101] also observed a significant decrease in naïve-like gene expression in peripheral CD8^+^ T cells after ICB, such as *TCF7*, *SELL*, *IL7R*, and *CCR7*. Collectively, scRNA-seq reveals that CD8^+^ T cells can shift from the naïve state to a more activated state during ICB, characterized by the expansion of effector cells and increased cytotoxic activities against cancer cells.

The dynamic changes in different subsets of exhausted CD8^+^ T cells during ICB vary among patients with different cancer types and TME compositions. Some scRNA-seq studies have reported the accumulation of progenitor exhausted T cells after ICB, while others have found the accumulation of terminally exhausted T cells. Liu et al. [102] found that *GZMK*^+^ precursor exhausted T (Texp) cells upregulated the expression of *CXCL13* and significantly accumulated after ICB in patients with NSCLC. Khan et al. [103] found that after PD-1 inhibition, Tex^prog^ cells differentiated into intermediate Tex (Tex^inter^) cells in anti-PD-1 responsive GL261 glioblastoma models. Kwon et al. [104] collected tumor tissues from patients with MSI-H gastric cancer who received ICB and conducted scRNA-seq analysis. They found an increase in the proportions of transitory and terminally exhausted CD8^+^ T cells after ICB, which contributed to the enhancement of cytotoxic function. Bi et al. [105] investigated a human RCC cohort and found that a subset of CD8^+^ T cells with low expression of the activation marker 4-1BB (4-1BB-Lo) had high progenitor exhausted signatures at baseline. These 4-1BB-Lo cells were activated by ICB and converted from a progenitor exhausted state to a more terminally exhausted state after ICB. The above studies revealed different distribution patterns of exhausted CD8^+^ T cell subsets after ICB, including progenitor, intermediate, and terminally exhausted cells. Liu et al. [106] found that this might be explained by different levels of immunosuppression in different TMEs. Terminally exhausted CD8^+^ T cells are more likely to accumulate in tumors with higher levels of immunosuppression after ICB, such as squamous cell carcinoma (SCC), basal cell carcinoma (BCC), and RCC, while precursor-like CD8^+^ T cells tend to accumulate in tumors with lower levels of immunosuppression, such as NSCLC and melanoma [102, 106].

Apart from CD8^+^ T cells, the application of scRNA-seq also reveals the dynamic changes in other cell types during ICB. For CD4^+^ T cells, Liu et al. [106] observed an increase in Th1-like CXCL13^+^ CD4^+^ T cells following effective ICB treatment across multiple cancer types, indicating their role as tumor-reactive cells. Liu et al. [102] found a slight decrease in Tregs after responding to ICB in NSCLC, consistent with the immunosuppressive role of Tregs. Li et al. [107] also observed a decrease in Tregs and an increase in CD40^+^ B cells after responding to ICB in patients with dMMR/MSI-H CRC. Additionally, Bi et al. [105] studied macrophages in RCC patients and observed a significant increase in M1-like proinflammatory patterns after ICB, which also upregulated some immune checkpoint and anti-inflammatory genes, potentially leading to transient effects and the ultimate failure of ICB.

In summary, scRNA-seq can identify dynamic changes in cell numbers and transcriptional patterns of cells in the TME, especially CD8^+^ T cells, in animal models or human patients at different time points during ICB treatment. Studying the changes in these cells can enhance our understanding of the impact of ICB on the TME, elucidating the process of patients’ responses to ICB and revealing the mechanisms underlying ICB function.

## Discovering novel biomarkers of ICB with scRNA-seq

ScRNA-seq is a potent technology for investigating crucial cell clusters with varying expression profiles between responders and non-responders to cancer immunotherapy. Differentially expressed genes or cell subsets displaying high expression differences between responders and non-responders can be used as biomarkers. Recent research has aimed to identify new biomarkers to predict the efficacy of ICB using scRNA-seq, shedding light on specific cell populations that might be indicative of ICB responsiveness in both the tumor and peripheral blood (Table 1).

**Table 1:** Summary of studies using scRNA-seq to discover novel ICB biomarkers

Cancer Types	Biomarkers	Samples	Cell types	Cell numbers	Main findings	Immunotherapy	Location	Ref.
Melanoma	*TCF7* expression of CD8^+^ T cells	N = 48 melanoma tumor tissues	CD8^+^ T cells	6,350	Higher levels of TCF7 protein in CD8^+^ T cells at baseline predict better responses to ICB therapy.	Anti-PD-1, anti-CTLA-4	Tumor	[108]
TNBC	CXCL13^+^ CD8^+^ T cells, CXCL13^+^ CD4^+^ T cells, and MMP9^+^ macrophages	N = 78 TNBC tumor tissues and blood samples	CD45^+^ cells	489,490	The baseline levels of CXCL13^+^ CD8^+^ T cells, CXCL13^+^ CD4^+^ T cells, and MMP9^+^macrophages are high in patients responding to PD-L1 blockade immunotherapy.	Atezolizumab	Tumor	[109]
NSCLC	PD1^+^ CXCL13^+^ T cells, IgG^+^ plasma cells, and SPP1^+^ macrophages	N = 35 NSCLC samples and N = 29 matched normal tissues	CD45^+^ cells	361,929	The lung cancer activation module (LCAM) includes PD1^+^ CXCL13^+^ T cells, IgG^+^ plasma cells, and SPP1^+^ macrophages. A high baseline LCAM score is a biomarker of a favorable ICB response.	Atezolizumab	Tumor	[110]
GC	CXCL13 expression in CD8^+^/CD4^+^ T cells	N = 19 GC samples and N = 19 normal tissues	T cells	53,768	CXCL13^+^ CD8^+^ and CD4^+^ T cells are important for TLS formation and high CXCL13 expression is associated with improved efficacy of ICB.	Pembrolizumab	Tumor	[111]
NSCLC	Tissue-resident memory CD8^+^ T (T_RM_)-like cells	N = 10 NSCLC patients	CD45^+^ cells	31,887	The high expression signature of CD8^+^ ITGAE^+^ T_RM_-like cells with high cytotoxicity, effector signature, and memory signature is predictive of favorable anti-PD-1 therapy responses.	Anti-PD-1	Tumor	[129]
Melanoma and NSCLC	*TOX* expression of T cells	N = 17 melanoma tumors and N = 14 NSCLC tumors	CD8^+^ T cells	3,195	*TOX* is specifically expressed by T cells and is a key transcription factor regulating T cell exhaustion. Lower expression of *TOX* in tumor-infiltrating T cells predicts a better response to anti-PD-1 therapy.	Nivolumab, pembrolizumab	Tumor	[113]
Multiple cancers	T_STR_ cells	N = 486 samples	T cells	308,048	T_STR_ cells exhibited high expression of heat-shock genes and stress response signatures. T_STR_ cells are more abundant in non-responsive tumors.	Anti-PD-1, anti-PD-L1, anti-CTLA-4	Tumor	[114]
Bladder cancer	Cytotoxic CD4^+^ T cells	N = 7 bladder cancer patients	CD4^+^ T cells	19,842	The signature genes of cytotoxic CD4^+^ T cells include *ABCB1*, *APBA2*, *SLAMF7*, *GPR18*, and *PEG10*. High expression of this signature is associated with a positive response to PD-1 blockade.	Atezolizumab	Tumor	[130]
NSCLC	Plasma cells	N = 44 NSCLC patients	CD79A^+^ B cells	20,362	High levels of intratumoral plasma cells at baseline predict a favorable response to PD-L1 blockade therapy.	Atezolizumab	Tumor	[115]
Melanoma	CD69^+^ B cells and IGLL5^−^ CD69^+^ B cells	N = 43 melanoma tumor tissues	B cells	N.A.	The fractions of CD69^+^ B cells and IGLL5^−^ CD69^+^ B cells at baseline are both higher in responders to ICB therapy. And a higher expression level of the TLS signature predicts a better prognosis after ICB.	Anti-PD-1, anti-CTLA-4	Tumor	[116]
NSCLC	FCRL4^+^ FCRL5^+^ memory B cells and CD16^+^ CX3CR1^+^ monocytes	N = 15 NSCLC tumor tissues	All the cells	92,330	High signatures of FCRL4^+^ FCRL5^+^ B cells and CD16^+^ CX3CR1^+^ monocytes in the TME predict a positive response to ICB therapy.	Anti-PD-1	Tumor	[131]
Melanoma	TREM2^high^ macrophages	N = 48 melanoma tumor tissues	CD45^+^ cells	16,291	High levels of TREM2^high^ macrophages at baseline are associated with poor clinical responses to ICB therapy.	Anti-PD-1, anti-CTLA-4	Tumor	[117]
NSCLC	TREM2^+^ TAMs	N = 26 NSCLC tumor tissues and blood samples	All the cells	50,483	High levels of TREM2^+^ TAMs at baseline predict poor clinical responses to immunotherapy.	Anti-PD-1	Tumor	[118]
HCC	APOC1	N = 8 HCC tumor and normal tissues and blood samples	CD45^+^ cells	212,494	APOC1 is over-expressed in TAMs of HCC tissues and is negatively correlated with PD-1 and PD-L1 expression. High levels of APOC1 predict unfavorable responses to anti-PD-1 therapy.	Anti-PD-1	Tumor	[119]
PDAC	LRRC15^+^ CAFs	N = 20 normal and PDAC mice, N = 22 PDAC patients	Dataset1: PDPN^+^ stromal cells Dataset2: CAFs	Dataset1: 13,454 Dataset2: 8,931	TGF-β driven LRRC15^+^ CAFs increase with tumor progression and higher expression of LRRC15^+^ CAFs signature is predictive of worse clinical response to atezolizumab in several human cancers.	Atezolizumab	Tumor	[85]
Breast cancer	ecm-myCAFs and TGFβ-myCAFs	N = 7 breast cancer tissues	FAP^hi^ CD29^med − hi^ SMA^hi^ CAFs	18,296	CAF subsets with high expression of genes encoding extracellular matrix proteins and TGF-β signaling pathway are respectively termed ecm-myCAFs and TGFβ-myCAFs. High levels of ecm-myCAFs and TGFβ-myCAFs predict primary resistance to ICB in several cancer types.	Pembrolizumab, nivolumab	Tumor	[86]
PDAC	meCAFs	N = 16 PDAC tumor tissues and normal pancreatic samples	CAFs	8,439	A new CAF subset exhibiting highly activated metabolic signatures and high expression of *PLA2G2A* and *CRABP2* is termed meCAFs. High levels of meCAFs at baseline predict a positive response to immunotherapy.	Anti-PD-1	Tumor	[120]
HNSCC	tsCAFs	N = 8 HNSCC tumor tissues	CAFs	5,414	Two CAF subsets (HNCAF-0/3) that can prevent exhaustion and enhance the cytotoxicity of CD8^+^ T cells are together termed T-cell stimulating CAF (tsCAF). High levels of tsCAF signatures at baseline predict favorable responses to ICB therapy.	Nivolumab, pembrolizumab	Tumor	[121]
Multiple cancers	TaNK cells	N = 1,223 samples	NK cells	160,011	An NK cell subset (CD56^dim^CD16^hi^ c6-DNAJB1) with low cytotoxicity and high stress response is termed tumor-associated NK (TaNK) cells. TaNK cell signatures predict resistance to ICB.	N.A.	Tumor	[122]
Melanoma	*SIRPA* expression of melanoma cells	N.A.	All the cells	N.A.	Melanoma cells interact with CD8^+^ T cells via the SIRPα/CD47 axis to increase CD8^+^ T cell cytotoxicity. Higher expression of *SIRPA* is predictive of a better response to ICB therapy in melanoma.	Anti-PD-1, anti-PD-L1	Tumor	[123]
Melanoma	Toxicity and large count number of CD8^+^ T cells	N = 16 blood samples	CD8^+^ T cells	22,445	Patients whose CD8^+^ T cells have lower toxicity and fewer expanded clones may have worse responses to ICB therapy.	Pembrolizumab, nivolumab, ipilimumab	Blood	[124]
Melanoma	MAIT cells	N = 183 blood samples from 20 patients	CD3^+^ CD8^+^ T cells	51,701	A high proportion of MAIT cells in peripheral blood at baseline predicts a favorable response to anti-PD-1 therapy.	Nivolumab, pembrolizumab	Blood	[125]
Melanoma	S100A9^+^ monocytes	N = 16 blood samples	PBMCs	~ 50,000	High levels of baseline S100A9^+^ monocytes in peripheral blood predict poor clinical responses to ICB therapy.	Nivolumab	Blood	[127]
Melanoma	CD8^+^ T_OXPHOS_ cells	N = 16 melanoma tumor tissues and blood samples	CD8^+^ T cells	173,061	A unique CD8^+^ T cell subset with high levels of oxidative phosphorylation (OXPHOS), high exhaustion markers, and high cytotoxicity markers is termed CD8^+^ T_OXPHOS_. High CD8^+^ T_OXPHOS_ cell signatures in tumors and peripheral blood predict ICB resistance.	N.A.	Blood and tumor	[126]
CRC	Inflammatory conditions and neutrophil-to-lymphocyte ratio (NLR)	N = 6 MSI-H CRC tumor samples	All the cells	12,118	Neutrophils participate in immunosuppression via the CD80/CD86-CTLA4 axis. A high NLR is predictive of an unfavorable ICB response in MSI-H CRC patients.	Anti-PD-1	Blood and tumor	[128]

TCF7, transcription factor 7; TNBC, triple-negative breast cancer; CXCL13, C-X-C motif chemokine ligand 13; MMP9, matrix metallopeptidase 9; NSCLC, non-small cell lung cancer; SPP1, secreted phosphoprotein 1; GC, gastric cancer; TLS, tertiary lymphoid structure; ITGAE, integrin subunit alpha E; TOX, thymocyte selection-associated high mobility group box; IGLL5, immunoglobulin lambda like polypeptide 5; FCRL4, Fc receptor-like 4; FCRL5, Fc receptor-like 5; CX3CR1, C-X3-C motif chemokine receptor 1; TREM2, triggering receptor expressed on myeloid cells 2; TAMs, tumor-associated macrophages; HCC, hepatocellular carcinoma; APOC1, apolipoprotein C1; PDAC, pancreatic ductal adenocarcinoma; LRRC15, leucine rich repeat containing 15; CAFs, cancer-associated fibroblasts; PDPN, podoplanin; TGF-β, transforming growth factor-β; FAP, fibroblast activation protein; SMA, smooth-muscle α actin; HNSCC, head and neck squamous cell carcinoma; SIRPA, signal regulatory protein α; MAIT cells, mucosal-associated invariant T cells; S100A9, S100 calcium-binding protein A9; PBMCs, peripheral blood mononuclear cells; CRC, colorectal cancer; MSI-H, microsatellite instability-high

### Biomarkers in the tumor

#### T cells

CD8^+^ T cells can directly kill cancer cells, and their expression patterns can determine the effectiveness of ICB therapy. Sade-Feldman et al. [108] identified two CD8^+^ T cell states in melanoma by scRNA-seq. One cluster, CD8_G, was enriched for genes related to memory and activation, including *TCF7* and *IL7R*, and was more abundant in ICB responders. They examined the expression levels of *TCF7* in CD8^+^ T cells and found more tumor-infiltrating TCF7^+^ CD8^+^ T cells in ICB responders and more TCF7^−^ CD8^+^ T cells in non-responders, demonstrating the predictive ability of *TCF7* expression of CD8^+^ T cells. Consistent with this study, other scRNA-seq studies also found that responders had higher levels of stem or progenitor T cells at baseline, whereas non-responders tended to accumulate more terminally exhausted T cells. Zhang et al. [109] applied scRNA-seq in TNBC tumors and discovered two clusters of CXCL13^+^ T cells (CD8-CXCL13 and CD4-CXCL13) with high expression of *CXCL13* and exhaustion genes *PDCD1*, *TIGIT*, and *CTLA-4*. Compared with non-responders, CD8-CXCL13 and CD4-CXCL13 were enriched at baseline and expanded after ICB in responders, indicating their tumor-reactive roles. They discovered a strong interaction between CXCL13^+^ T cells and follicular B cells via the CXCL13-CXCR5 axis, which enhanced the anti-tumor activity, consistent with other scRNA-seq studies [110, 111]. In addition, another study showed that CXCL13 was a B cell chemoattractant and was associated with the formation of tertiary lymphoid structures (TLSs), the ectopic lymphoid tissues that form around tumors and participate in anti-tumor immune responses [112]. Liu et al. [106] demonstrated that CXCL13 was a marker of tumor-reactive T cells. Measuring the abundance of CXCL13^+^ CD8^+^ and CXCL13^+^ CD4^+^ T cells together in tumor samples is a powerful biomarker of response to ICB in multiple cohorts, with higher predictive accuracy than TMB [106]. Kim et al. [113] classified CD8^+^ T cells into *PDCD1*-high and *PDCD1*-low subsets based on *PDCD1* expression levels from scRNA-seq datasets of both melanoma and NSCLC. They found that the transcription factor TOX was a crucial promoter regulating T cell exhaustion. Additionally, low baseline expression of *TOX* in tumor-infiltrating T cells was associated with a positive ICB response, suggesting the predictive ability of *TOX*. These studies highlight the importance of baseline accumulation of stem/memory-like or progenitor exhausted cells for a favorable response to ICB, as opposed to terminally exhausted CD8^+^ T cells. A pan-cancer scRNA-seq study [114] discovered a novel T-cell state characterized by high expression of *HSPA1A* and *HSPA1B* as well as a stress response signature. This CD8^+^/CD4^+^ T_STR_ group was found to be predictive of an unfavorable response to ICB across multiple cancer types, providing a new T-cell biomarker different from T-cell exhaustion.

#### B cells

B cells within the TME are another group of immune cells crucial for robust anti-tumor immunity. Patil et al. [115] clustered B cells from scRNA-seq data of NSCLC patients and discovered a plasma cell subcluster with signature genes, including *MZB1*, *DERL3*, *JSRP1*, and *IGHG2*. They demonstrated that a high plasma cell signature in the TME could predict a favorable response to ICB in multiple cohorts. Cabrita et al. [116] studied B cells using a melanoma scRNA-seq dataset and noticed that baseline CD69^+^ B cells and IGLL5^−^ CD69^+^ B cells were both more abundant in responders. They also discovered that B-cell-rich tumors had a higher proportion of *TCF7*- or *IL7R*-expressing naïve or memory-like CD8^+^ and CD4^+^ T cells than B-cell-poor tumors, which suggested that a higher level of tumor-infiltrating B cells could recruit more progenitor T cells to TLSs and potentially lead to a better response to ICB.

#### TAMs

TAMs can serve as another type of biomarker for predicting ICB response, as M1- and M2-type macrophages play distinct roles in the TME. Xiong et al. [117] identified a macrophage subset primarily in melanoma non-responders with high expression of *TREM2*, M2-like signatures, and genes associated with the complement system and complement cascade activation. The complement system associated with this TREM2^high^ macrophage subset could alter the composition of the TME and contribute to ICB resistance. Consistent with Xiong et al. [117], a study by Zhang et al. [118] also found a TREM2^+^ TAM subset from scRNA-seq data of NSCLC and demonstrated its immunosuppressive role in the TME. This TREM2^+^ cluster was enriched for genes linked to pro-tumor pathways and could strongly interact with FOXP3^+^ Tregs through the IL1B/IL1R interaction, suppressing the anti-tumor effects. These studies revealed that high infiltration of TREM2^+^ TAMs might predict a poor response to ICB in various cancers. In addition, Hao et al. [119] identified a specific TAM subset expressing APOC1, FABP1, and C1QC in HCC patients. They discovered that APOC1 exhibited significantly higher expression levels in TAMs of HCC tissues than adjacent tissues or peripheral blood and was negatively correlated with PD-1 and PD-L1 expression. Therefore, high expression of APOC1 in TAMs could predict an unfavorable ICB response in HCC patients. In the study by Zhang et al. [109], MMP9^+^ macrophages with high expression of *PLA2G2D*, *IL2RG*, and *CXCL9* were enriched in responders at baseline, suggesting their predictive value in TNBC patients receiving ICB therapy.

#### CAFs

CAFs interact with cancer cells and immune cells in the TME, impacting the responsiveness to ICB therapy. Dominguez et al. [85] analyzed stromal cells expressing podoplanin from the pancreases of normal mice and mice with PDAC. They identified a subcluster of myCAFs that showed enrichment for TGFβ gene signatures with significantly higher expression of *LRRC15* than natural fibroblasts. They demonstrated that high LRRC15^+^ CAF signatures could predict poor responses to atezolizumab in several types of human cancers. Similar to this finding, Kieffer et al. [86] discovered two subsets of myCAFs from breast cancer scRNA-seq data: ecm-myCAFs, which exhibited high expression of *LRRC15* and were associated with ECM remodeling, and TGFβ-myCAFs, which had high expression of *TGFβ1* and were associated with the TGF-β signaling pathway. These two subclusters might be linked to an immunosuppressive TME enriched for Tregs and deficient in CD8^+^ T cells. Non-responders with melanoma or NSCLC who received ICB had higher baseline expression levels of ecm-myCAFs and TGFβ-myCAFs. Wang et al. [120] identified a novel CAF subcluster termed meCAF that had a highly activated metabolic state with the marker genes *PLA2G2A* and *CRABP2* based on scRNA-seq data from PDAC. In loose-type PDAC with low desmoplasia, meCAFs were enriched and were associated with high metastatic potential and poor prognosis. However, patients with high meCAF infiltration at baseline showed a dramatic response to anti-PD-1 therapy, indicating that meCAFs could predict favorable responses to PDAC immunotherapy. Obradovic et al. [121] conducted scRNA-seq on HNSCC samples and identified five distinct CAF subclusters. In anti-PD-1 responders, the gene signatures of two CAF subsets (HNCAF-0 and HNCAF-3) were enriched at baseline and expanded after treatment. The researchers also demonstrated that HNCAF-0/3 could inhibit the exhaustion of CD8^+^ T cells and enhance their memory phenotypes and cytolytic function, as evidenced by functional assays. Thus, the gene signatures of HNCAF-0/3 were designated as T cell-stimulating CAF (tsCAF), which could predict the responsiveness to ICB. These studies provide additional evidence that CAFs in the TME are heterogeneous, comprising both pro- and anti-tumor subsets that can differentially regulate anti-tumor immunity and ultimately modulate the effects of ICB [82].

#### NK cells

As innate immune cells, NK cells also play an essential role in the anti-tumor response, and their heterogeneity has been revealed through single-cell technologies. In a pan-cancer scRNA-seq study [122], CD56^dim^CD16^hi^ c6-DNAJB1 NK cells were designated as tumor-associated NK (TaNK) cells, which were enriched in tumors and exhibited impaired cytotoxicity, indicative of a terminal state. The signatures of TaNK cells were more pronounced in non-responders than responders, implying their potential as an ICB biomarker.

#### Cancer cells

In addition to cells in the cancer stroma, the RNA expression patterns of cancer cells themselves may be indicative of ICB outcome. For example, Zhou et al. [123] analyzed several melanoma scRNA-seq datasets and revealed that melanoma cells had elevated expression levels of signal regulatory protein α (SIRPα, encoded by *SIRPA*) and could strongly interact with CD8^+^ T cells via the SIRPα/CD47 axis, thus enhancing the cytotoxicity of CD8^+^ T cells. ICB responders had higher *SIRPA* expression in melanoma cells than non-responders, demonstrating the predictive potential of *SIRPA* expression in melanoma cells.

### Biomarkers in peripheral blood

Although cellular components in the TME directly reflect factors that influence cancer cell growth, metabolism, and metastasis, obtaining these samples from patients requires invasive procedures. Therefore, it is essential to investigate circulating biomarkers to minimize the harm caused by biopsies. Watson et al. [124] used scRNA-seq and single-cell V(D)J sequencing to analyze peripheral CD8^+^ T cells collected before and after ICB therapy from melanoma patients. They found that patients with low cytotoxicity and small expanded clone numbers in their peripheral CD8^+^ T cells at baseline had poor clinical outcomes following ICB. De Biasi et al. [125] conducted scRNA-seq on circulating CD8^+^ T cells from patients with metastatic melanoma and found that mucosal-associated invariant T (MAIT) cells were present in a higher proportion and had higher activation signatures before and after ICB in responders. Moreover, CXCR4 expression on peripheral MAIT cells could mediate their migration to the site of metastatic tumors and exert their proinflammatory and cytotoxic functions, further supporting the predictive value of peripheral MAIT cells for melanoma ICB therapy. Li et al. [126] analyzed scRNA-seq data from melanoma patients and identified a subset of CD8^+^ T cells that exhibited high levels of oxidative phosphorylation (OXPHOS) metabolism and expression of exhaustion and cytotoxic signatures. This unique CD8^+^ T cell cluster was present in both tumors and peripheral blood and was termed CD8^+^ T_OXPHOS_ cells. Additionally, the researchers found that melanoma patients who showed resistance to ICB had higher levels of OXPHOS in CD8^+^ T_OXPHOS_ cells in both tumors and peripheral blood.

In addition to CD8^+^ T cells, other immune cells in peripheral blood have been shown to carry predictive potential in other studies using scRNA-seq. Pour et al. [127] analyzed peripheral blood mononuclear cells (PBMCs) from melanoma patients before and during nivolumab treatment. They identified a monocyte cluster expressing significantly higher levels of S100A9 and other S100A proteins in non-responders than in responders. Further validation showed that S100A9^+^ monocytes were significantly more abundant in non-responders in a melanoma cohort, suggesting its predictive potential for ICB. Sui et al. [128] analyzed scRNA-seq data from MSI-H CRC patients and discovered that neutrophils interacted with CD8^+^ T cells in the tumor via the CD80/CD86-CTLA4 axis, contributing to local inflammation that predicted an unfavorable response to ICB. Moreover, the inflammatory response was correlated with peripheral leukocytes, and a high neutrophil-to-lymphocyte ratio (NLR) could also predict a poor response to ICB.

## Applying scRNA-seq to identify novel therapeutic targets for immunotherapy

Due to the low response rate to ICB in cancer treatment, identifying novel therapeutic targets to combine with CTLA-4 or PD-1/PD-L1 inhibitors is essential for enhancing anti-tumor effects. Applying scRNA-seq enables the identification of critical molecules or cell subtypes that contribute to immunosuppressive tumor microenvironments and the failure of ICB therapy. To identify new treatment targets for immunotherapy, scRNA-seq can be employed to discover crucial genes expressed by cancer cells as well as other immunosuppressive cells, compare the expression of genes between responders and non-responders, evaluate changes in the TME after targeting therapeutic targets, and identify immunosuppressive intercellular interactions in the TME. Moreover, to strengthen the credibility of scRNA-seq results, it is necessary to confirm the efficacy of proposed therapeutic targets through functional assays, such as cell lines and animal models. The studies applying scRNA-seq to identify novel treatment targets for immunotherapy are summarized in Table 2.

**Table 2:** Summary of studies identifying new therapeutic targets for immunotherapy revealed by scRNA-seq

Cancer types	Cell types	Therapeutic targets	Effects	Therapeutic approach	Mechanisms	Ref.
Melanoma	Cancer cells	CDK4/6	Pro-tumor	CDK4/6 inhibitor (palbociclib and abemaciclib)	Cancer cell immune evasion and T cell exclusion	[132]
TNBC	Cancer cells	MUC1-C	Pro-tumor	MUC1-C inhibitor GO-203	Depletion and dysfunction of CD8^+^ T cells	[144]
PDAC	Cancer cells	SLC4A4	Pro-tumor	SLC4A4 inhibitor DIDS	A bicarbonate transporter increasing TME acidity	[133]
NPC	Cancer cells	CD70	Pro-tumor	Anti-CD70 antibody cusatuzumab	Promoting the suppressive function of Tregs via the CD70-CD27 axis	[143]
Melanoma	CD8^+^ T cells	NKG7	Anti-tumor	NKG7 mRNA transfection	Cytotoxic function in CD8^+^ T cells	[137]
COAD and RCC	CD8^+^ T cells	GITR	Anti-tumor	Agonist anti-GITR antibody	A costimulatory receptor	[145]
Melanoma and sarcoma	CD8^+^ T cells, CD4^+^ T cells	BHLHE40	Anti-tumor	–	A transcription factor required for effective ICB therapy	[146]
Glioma	Cancer cells/myeloid cells – CD8^+^ T cells	CLEC2D/CD161	Pro-tumor	Anti-CD161 antibody HP-3G10	Suppressing cytotoxicity and cytokine secretion of T cells	[147]
LR-CHL	CD4^+^ helper T cells – B cells	CXCL13/CXCR5	Pro-tumor	–	Preventing B cell activation and maturation	[148]
Ovarian cancer	NK cells, CD8^+^ T cells	BRD1	Pro-tumor	BRD1 inhibitor BAY-299	Negatively regulating the activity of T cells and NK cells	[149]
Multiple cancers	NK cells	HIF-1α	Pro-tumor	HIF-1α inhibitor	A transcription factor inhibiting the activation of NK cells	[150]
Multiple myeloma	NK cells	ZNF683	Pro-tumor	–	A transcription factor regulating NK cell exhaustion	[151]
Multiple cancers	CD4^+^ Tregs	CCR8	Pro-tumor	ADCC-prone anti-CCR8 nanobody	A marker induced by Treg activation	[152]
Multiple cancers	CD4^+^ Tregs	CD177	Pro-tumor	Anti-CD177 antibody MEM166	Mediating immune suppressive function of Tregs	[153]
ICC	CD4^+^ Tregs	MEOX1	Pro-tumor	–	A transcription factor regulating Treg hyperactivation	[135]
PDAC	T cells – cancer cells	CCL5/SDC1	Pro-tumor	–	Promoting tumor cell migration	[154]
Glioma	TAMs, CD4^+^ Tregs, CD8^+^ T cells	S100A4	Pro-tumor	–	A small calcium-binding protein related to immunosuppression	[134]
Multiple cancers	TAMs	TREM2	Pro-tumor	Anti-TREM2 monoclonal antibody	Associated with immunosuppression	[138]
Multiple cancers	TAMs	APOE	Pro-tumor	APOE inhibitor COG 133TFA	An oncomarker of TAMs associated with M2-TAMs recruitment	[139]
HCC	TAMs	SPP1	Pro-tumor	Anti-SPP1 monoclonal antibody	Inducing the formation of tumor immune barrier by interacting with CAFs	[155]
HCC	TAMs	PPT1	Pro-tumor	PPT1 inhibitor DC661	Associated with the immunosuppressive TME	[156]
GBM	TAMs	Siglec-9	Pro-tumor	–	Inhibiting the activation of CD8^+^ and CD4^+^ T cells	[157]
RCC	TAMs – cancer cells	IL-1β/IL-1R1	Pro-tumor	Anti-IL1β antibody	Mediating adaptive myeloid resistance and cancer cell EMT	[158, 159]
PDAC	CAFs	HIF2	Pro-tumor	HIF2 inhibitor PT2399	Mediating immunosuppression in the TME	[160]
reRCC	CAFs	Gal1	Pro-tumor	Gal1 inhibitor OTX008	An immunosuppressive factor triggering T cell apoptosis	[161]
HCC	CAFs	CD36	Pro-tumor	CD36 inhibitor sulfo-N-succinimidyl oleate (SSO)	Recruiting MDSCs to increase immunosuppression of the TME	[162]
ICC	CD146^+^ vCAFs – cancer cells	IL-6/IL-6R	Pro-tumor	IL-6R neutralizing antibody or IL-6/IL-6R signaling inhibitor	Enhancing ICC malignancy	[84]
Prostate cancer	Arterial TECs – tip TECs	CXCL12/CXCR4	Pro-tumor	CXCR4 inhibitor AMD3100	Triggering neovascularization	[142]
mUC and mRCC	Multiple cells	CXCL8/CXCR2	Pro-tumor	–	Cancer cell growth and metastasis	[136]
CRC	Multiple cells	CD73	Pro-tumor	CD73 inhibitor AB680	A checkpoint enzyme converting extracellular ATP to immunosuppressive adenosine	[140]
Melanoma and myeloma	Multiple cells	GCN2	Pro-tumor	GCN2 inhibitor GCN2iB	Suppressing inflammatory macrophage polarization and promoting cancer cell survival	[163, 164]
Bladder cancer	Multiple cells	CD39	Pro-tumor	CD39 inhibitor sodium polyoxotungstate	Associated with immunosuppression	[141]
Multiple cancers	Multiple cells	IGSF9	Pro-tumor	Anti-IGSF9 antibody	Suppressing T cell activation	[165]

CDK4/6, cyclin-dependent kinase 4/6; TNBC, triple-negative breast cancer; MUC1-C, mucin 1-C type protein; PDAC, pancreatic ductal adenocarcinoma; SLC4A4, solute carrier family 4 member 4; DIDS, 4,4′-diisothiocyano-2,2′-stilbenedisulfonic acid; TME, tumor microenvironment; NPC, nasopharyngeal carcinoma; Tregs, regulatory T cells; NKG7, natural killer cell granule protein-7; COAD, colon adenocarcinoma; RCC, renal cell carcinoma; GITR, glucocorticoid-induced tumor necrosis factor receptor-related protein; BHLHE40, basic helix-loop-helix family member E40; ICB, immune checkpoint blockade; CLEC2D, C-type lectin domain family 2 member D; LR-CHL, lymphocyte-rich classic Hodgkin lymphoma; CXCL13, C-X-C motif chemokine ligand 13; CXCR5, C-X-C chemokine receptor type 5; NK cells, natural killer cells; BRD1, bromodomain-containing protein 1; HIF-1α, hypoxia inducible factor 1 subunit alpha; ZNF683, zinc finger protein 683; CCR8, CC chemokine receptor 8; ADCC, antibody-dependent cell-mediated cytotoxicity; ICC, intrahepatic cholangiocarcinoma; MEOX1, mesenchyme homeobox 1; CCL5, C-C motif chemokine ligand 5; SDC1, syndecan-1; TAMs, tumor-associated macrophages; S100A4, S100 calcium-binding protein A4; TREM2, triggering receptor expressed on myeloid cells 2; APOE, apoprotein E; HCC, hepatocellular carcinoma; SPP1, secreted phosphoprotein 1; PPT1, palmitoylprotein thioesterase 1; GBM, glioblastoma; Siglec-9, sialic acid-binding Ig-like lectin 9; IL-1β, interleukin-1 beta; IL-1R1, interleukin-1 receptor type 1; EMT, epithelial-mesenchymal transition; CAFs, cancer-associated fibroblasts; HIF2, hypoxia-inducible factor-2; reRCC, recurrent renal cell carcinoma; Gal1, Galectin-1; MDSCs, myeloid-derived suppressor cells; IL-6, interleukin-6; IL-6R, IL-6 receptor; TECs, tumor endothelial cells; CXCL12, C-X-C motif chemokine ligand 12; CXCR4, C-X-C chemokine receptor type 4; mUC, metastatic urothelial carcinoma; mRCC, metastatic renal cell carcinoma; CXCL8, C-X-C motif chemokine ligand 8; CXCR2, C-X-C chemokine receptor type 2; CRC, colorectal cancer; GCN2, general control nonderepressible 2; IGSF9, immunoglobulin superfamily member 9

By utilizing scRNA-seq, it is possible to identify immune-evasion genes expressed by cancer cells that can lead to the failure of ICB. Targeting these genes represents a potential approach to enhancing the efficacy of ICB treatment. Jerby-Arnon et al. [132] studied malignant cells from melanoma patients using scRNA-seq and identified an immune resistance program including cyclin-dependent kinase 4 (*CDK4*). Combining CDK4/6 inhibitors and ICB enhanced the response to therapy. Cappellesso et al. [133] performed scRNA-seq on PDAC patients and identified *SLC4A4* as the most abundantly expressed bicarbonate transporter gene of PDAC epithelial cells. Inhibition of SLC4A4 reduced the acidification of the TME and repressed tumor growth when combined with ICB, confirming its potential as a therapeutic target.

Some genes expressed by immunosuppressive cells in the TME revealed by scRNA-seq can also be therapeutic targets. Abdelfattah et al. [134] observed high expression of *S100A4* in exhausted T cells, Tregs, and pro-tumor myeloid cells by scRNA-seq of human gliomas and found that it was associated with a poor prognosis. Further functional assays confirmed the immunosuppressive role of S100A4, indicating its potential as a therapeutic target. Alvisi et al. [135] found increased expression of MEOX1 in tumor-infiltrating Tregs by scRNA-seq data of ICC and demonstrated that the transcription factor MEOX1 could reprogram peripheral Tregs toward a tumor-infiltrating immunosuppressive signature.

Genes showing differential expression between responders and non-responders, as discovered by scRNA-seq, may serve as potential treatment targets to improve ICB efficacy. Yuen et al. [136] found that baseline *IL8* (*CXCL8*) expression in peripheral myeloid cells was higher in non-responders, according to scRNA-seq data from PBMCs of patients with metastatic urothelial carcinoma and metastatic renal cell carcinoma. *IL8*-high cells downregulated genes related to antigen presentation, contributing to immunosuppression. Therefore, blocking CXCR2, the receptor of IL-8, could be an effective strategy to sensitize tumors to PD-1 inhibitors. Wen et al. [137] analyzed peripheral CD8^+^ T cells from melanoma patients receiving ICB and found that *NKG7* was upregulated in responders and downregulated in non-responders after ICB treatment. The researchers confirmed the cytolytic function of NKG7 through functional assays and demonstrated that combining *NKG7* mRNA therapy and ICB enhanced the cytotoxicity of CD8^+^ T cells.

Using scRNA-seq to study changes in the TME after targeting certain genes or proteins can evaluate their potential as therapeutic targets. Molgora et al. [138] observed that in mouse models, the combination of anti-TREM2 treatment of TAMs and anti-PD-1 treatment remodeled the landscape of macrophages in the TME, with a decrease in MRC1^+^ (CD206^+^) macrophages and an increase in immunostimulatory iNOS^+^ macrophages. The researchers highlighted the crucial role of TREM2 in immunotherapy resistance, consistent with the findings of Xiong et al. [117] and Zhang et al. [118]. Hui et al. [139] performed scRNA-seq on tumor-bearing mice deficient in APOE and noticed a decrease in M2-like C1QC^+^ and CCR2^+^ TAMs, suggesting that targeting APOE^+^ macrophages could be a novel strategy to combine with ICB for cancer treatment. Kim et al. [140] conducted scRNA-seq analysis to compare the effects of CD73 inhibitors and PD-1 inhibitors on tumor-bearing mice with CRC. They discovered that CD73 inhibition improved the anti-tumor functions of Tregs and exhausted CD8^+^ T cells distinctly from PD-1 inhibition, showing that the combination of CD73 inhibitors and PD-1 inhibitors could synergistically kill cancer cells in CRC treatment. Liu et al. [141] showed that inhibiting CD39 in bladder cancer increased tumor-infiltrating NK cells and conventional type 1 dendritic cells (cDC1) and enhanced the cytotoxicity of CD8^+^ T cells. They also found that CD39 inhibition had synergistic effects with cisplatin but not with PD-1 inhibitors, indicating its potential for combination with chemotherapy rather than immunotherapy.

ScRNA-seq analysis can also effectively capture cellular interactions within the TME, revealing the ligand-receptor pairs that may serve as promising targets for immunotherapy interventions. Zhang et al. [84] applied scRNA-seq in ICC samples and identified an interaction between CD146^+^ vascular CAFs (vCAFs) and malignant cells via the IL-6/IL-6R axis, which could promote tumorigenesis and cancer stemness. Inhibiting the IL-6/IL-6R axis reduced tumor progression, indicating its potential as a treatment target. Heidegger et al. [142] focused on tumor endothelial cells (TECs) in prostate cancer patients using scRNA-seq data. They identified an interaction between arterial TECs and tip TECs mediated by the CXCL12/CXCR4 axis, which could promote angiogenesis. The CXCR4 inhibitor AMD3100 could target this ligand-receptor pair and reduce the number and density of vessels. Gong et al. [143] analyzed scRNA-seq data and found that nasopharyngeal carcinoma cells interacted with Tregs via the CD70-CD27 axis, thereby enhancing the proliferation and suppressive function of Tregs. CD70 blockade could be synergistic with PD-1 inhibition to enhance the anti-tumor response of T cells.

## Conclusions and further perspectives

Over the past decade, the use of ICB in cancer treatment has proven successful in delaying tumor progression and prolonging overall survival in some patients. However, this therapy only benefits a small proportion of individuals with specific cancer types. The intratumoral and intertumoral heterogeneity of the TME is a key factor that contributes to differences in responsiveness to ICB. As an emerging high-throughput technology, scRNA-seq is increasingly being used to study the heterogeneous transcriptomic information of individual cells in the TME and identify crucial cell subsets involved in ICB responsiveness from a large amount of bioinformatic data. In recent years, many studies have employed scRNA-seq to investigate the mechanisms underlying responsiveness to ICB in multiple cancers, in both animal models and humans. By applying scRNA-seq to tumor samples and PBMCs, researchers can capture the dynamic changes in crucial cell subsets during ICB, identify biomarkers that can predict response to ICB, and discover novel therapeutic targets in the TME for immunotherapy.

To precisely capture the dynamic alterations of cell subsets and obtain convincing results, it may be helpful to take tumor samples from patients in a large cohort at multiple time points during ICB. However, the recruitment of enough patients for scRNA-seq is difficult, and the repeated procedures necessary to obtain multiple samples can cause harm to patients. To solve this problem, some studies have conducted scRNA-seq on animal models and validated the conclusions in human cohorts. In addition, investigating key cell subclusters in peripheral blood, which is noninvasive to patients, provides an alternative approach to understanding the mechanisms of ICB response or resistance.

However, the reliability of scRNA-seq analysis is limited not only by the number of cells collected from patients but also by the inherent shortcomings of the technology, such as single-cell isolation, library amplification, technical noise, and sequencing depth and quality [166]. In addition, since scRNA-seq can only detect RNA expression levels and cannot directly capture protein expression, it is often used in combination with other technologies, such as flow cytometry, mass spectrometry, and cytometry by time-of-flight (CyToF) to obtain transcriptomic and proteomic data of individual cells. Another single-cell technology, named cellular indexing of transcriptomes and epitopes by sequencing (CITE-seq), has been applied in many studies. It combines scRNA-seq with cell surface protein detection, enabling the simultaneous profiling of the single-cell transcriptome and cell surface proteome [167]. Furthermore, although single-cell technologies capture biological information at the single-cell level, they do not preserve the spatial information of cells’ original locations since cells are dissociated from tissues. By integrating single-cell technologies with spatial omics technologies, such as spatial transcriptomes or spatial proteomics, we can both map the cellular phenotypes at a high resolution and reflect the architecture of the tissue microenvironment, allowing us to have a deep understanding of the TME heterogeneity [168]. In future studies, it would be beneficial to combine scRNA-seq with these techniques to more precisely characterize cellular profiles in large cohorts of cancer patients. Additionally, confirming scRNA-seq findings through functional assays would provide greater confidence in the results. These insights potentially inform clinical practice for precision medicine by identifying critical cell subsets involved in ICB responsiveness, improving patient stratification strategies, and searching for novel therapeutic targets.

## Data Availability

Not applicable.

## Abbreviations

ICB: Immune checkpoint blockade
TME: Tumor microenvironment
scRNA-seq: Single-cell RNA sequencing
ICIs: Immune checkpoint inhibitors
PD-1: Programmed cell death 1
PD-L1: Programmed cell death 1 ligand 1
CTLA-4: Cytotoxic T lymphocyte-associated protein 4
dMMR: Mismatch repair deficiency
MSI-H: Microsatellite instability-high
TMB: Tumor mutational burden
ECM: Extracellular matrix
RNA-seq: RNA sequencing
BCLC: Barcelona Clinic Liver Cancer
HCC: Hepatocellular carcinoma
APCs: Antigen-presenting cells
FDA: Food and Drug Administration
TCR: T cell receptor
RCC: Renal cell carcinoma
TNBC: Triple-negative breast cancer
NSCLC: Non-small cell lung carcinoma
HNSCC: Head and neck squamous cell carcinomas
MCC: Merkel cell carcinoma
LAG-3: Lymphocyte-activation gene 3
TIM-3: T-cell immunoglobulin and mucin-domain 3
TIGIT: T cell immunoreceptor with Ig and ITIM domains
Tregs: Regulatory T cells
Th1 cells: T helper 1 cells
NK cells: Natural killer cells
MHC: Major histocompatibility complex
HLA-I: Human leukocyte antigen class I
ctDNA: Circulating tumor DNA
DCs: Dendritic cells
NKT cells: Natural killer T cells
γδT cells: gamma delta T cells
MDSCs: Myeloid-derived suppressor cells
TAMs: Tumor-associated macrophages
TANs: Tumor-associated neutrophils
Bregs: Regulatory B cells
CAFs: Cancer-associated fibroblasts
IL-2: Interleukin-2
TGF-β: Transforming growth factor-β
ROS: Reactive oxygen species
iNOS: Nitric oxide synthase
VEGF: Vascular endothelial growth factor
MMP: Matrix metalloprotein
PDAC: Pancreatic ductal adenocarcinoma
MMPs: Matrix metalloproteinases
CXCL12: C-X-C motif chemokine ligand 12
CCL2: C-C motif chemokine ligand 2
iCAFs: inflammatory CAFs
myCAFs: myofibroblastic CAFs
ECs: Endothelial cells
TCF1: Transcription factor 1
TCR-seq: TCR sequencing
SCC: Squamous cell carcinoma
BCC: Basal cell carcinoma
TLSs: Tertiary lymphoid structures
MAIT cells: Mucosal-associated invariant T cells
OXPHOS: Oxidative phosphorylation
PBMCs: Peripheral blood mononuclear cells
NLR: Neutrophil-to-lymphocyte ratio
CDK4: Cyclin-dependent kinase 4
cDC1: Conventional type 1 dendritic cells
TECs: Tumor endothelial cells
vCAFs: vascular CAFs
CyToF: Cytometry by time-of-flight
CITE-seq: Cellular indexing of transcriptomes and epitopes by sequencing
